# Complete DNA sequences of the plastid genomes of two parasitic flowering plant species, *Cuscuta reflexa *and *Cuscuta gronovii*

**DOI:** 10.1186/1471-2229-7-45

**Published:** 2007-08-22

**Authors:** Helena T Funk, Sabine Berg, Karin Krupinska, Uwe G Maier, Kirsten Krause

**Affiliations:** 1 Department of Cell Biology, Philipps-University Marburg, Karl-von-Frisch-Str., D-35032 Marburg, Germany; 2 Botanisches Institut, Christian-Albrechts-Universität Kiel, Olshausenstr. 40, D-24098 Kiel, Germany; 3 Institutt for Biologi, Universitetet i Tromsø, 9037 Tromsø, Norway

## Abstract

**Background:**

The holoparasitic plant genus *Cuscuta *comprises species with photosynthetic capacity and functional chloroplasts as well as achlorophyllous and intermediate forms with restricted photosynthetic activity and degenerated chloroplasts. Previous data indicated significant differences with respect to the plastid genome coding capacity in different *Cuscuta *species that could correlate with their photosynthetic activity. In order to shed light on the molecular changes accompanying the parasitic lifestyle, we sequenced the plastid chromosomes of the two species *Cuscuta reflexa *and *Cuscuta gronovii*. Both species are capable of performing photosynthesis, albeit with varying efficiencies. Together with the plastid genome of *Epifagus virginiana*, an achlorophyllous parasitic plant whose plastid genome has been sequenced, these species represent a series of progression towards total dependency on the host plant, ranging from reduced levels of photosynthesis in *C. reflexa *to a restricted photosynthetic activity and degenerated chloroplasts in *C. gronovii *to an achlorophyllous state in *E. virginiana*.

**Results:**

The newly sequenced plastid genomes of *C. reflexa *and *C. gronovii *reveal that the chromosome structures are generally very similar to that of non-parasitic plants, although a number of species-specific insertions, deletions (indels) and sequence inversions were identified. However, we observed a gradual adaptation of the plastid genome to the different degrees of parasitism. The changes are particularly evident in *C. gronovii *and include (a) the parallel losses of genes for the subunits of the plastid-encoded RNA polymerase and the corresponding promoters from the plastid genome, (b) the first documented loss of the gene for a putative splicing factor, MatK, from the plastid genome and (c) a significant reduction of RNA editing.

**Conclusion:**

Overall, the comparative genomic analysis of plastid DNA from parasitic plants indicates a bias towards a simplification of the plastid gene expression machinery as a consequence of an increasing dependency on the host plant. A tentative assignment of the successive events in the adaptation of the plastid genomes to parasitism can be inferred from the current data set. This includes (1) a loss of non-coding regions in photosynthetic *Cuscuta *species that has resulted in a condensation of the plastid genome, (2) the simplification of plastid gene expression in species with largely impaired photosynthetic capacity and (3) the deletion of a significant part of the genetic information, including the information for the photosynthetic apparatus, in non-photosynthetic parasitic plants.

## Background

Parasitism among land plants has evolved independently in a variety of angiosperm families. Although knowledge of their biology is still rudimentary and limited to a relatively small number of species, it has nevertheless become apparent that a great diversity exists with respect to the anatomical and physiological adaptation to a parasitic lifestyle and the nutritional dependence on the host plants [1].

The parasitic genus *Cuscuta *comprises a range of species with different degrees of adaptation to the parasitic lifestyle. While all species have in common that they contain neither leaves nor roots and obtain both organic and inorganic nutrients in addition to water from their host plant through haustoria, there is some variation with respect to the structure and function of the plastids. While in some species thylakoids and even grana stacks are still present and the accumulation of photosynthetic pigments has been observed, many of the *Cuscuta *species contain plastids with a strongly reduced thylakoid system [2]. These species accumulate comparatively small amounts of chlorophyll. The chlorophyll content and photosynthetic activity are influenced by external factors such as nutrient supply, light intensity and the host plant species [2,3]. However, the net CO_2 _fixation rate never exceeds the compensation point [1,2,4] such that all *Cuscuta *species are placed within the group of holoparasitic plants.

Loss of photosynthesis may directly influence the gene content of the plastid genome in parasitic plants. While no comprehensive effort has so far been undertaken to identify nuclear-encoded plastid proteins in *Cuscuta *or other parasitic plants, the plastid genome and its coding capacity has been under investigation in a number of parasitic plants. Here, losses of genes have been reported for several species, including *Cuscuta reflexa *[5-7], *Conopholis americana *[8], *Orobanche hederae *[9] and *Epifagus virginiana *[10]. Especially in the case of *Cuscuta*, where photosynthesis activity ranges from reduced levels to a non-photosynthetic status [2], differential gene losses from the plastid genome must be expected. Under the assumption that a correlation exists between genome structure and gene content, first hints for genomic adaptations to holoparasitism were seen in hybridization studies on *Cuscuta *plastid DNA, in which differences in the genome sizes correlate with photosynthetic capacity [11,12].

Compared to plastid gene expression in green algae, land plant plastids exhibit several differences. These include the transcription mechanisms of plastid genes, intron splicing as well as RNA editing. Contrary to algae, land plant plastid chromosomes are transcribed by two different RNA polymerases. Beside the plastid-encoded RNA polymerase (PEP) that is thought to be mainly responsible for the expression of the components of the photosynthetic apparatus and that is present in algae as well, a nuclear-encoded RNA polymerase (NEP) additionally acts in land plant plastids. The main activity of the NEP seems to be the expression of housekeeping genes [13,14].

Introns and RNA editing are common in land plant chloroplasts which distinguish them further from green algal chloroplasts. Typically, one group I intron and about 20 group II introns are present in the plastid genome of photosynthetic land plants [15]. Chloroplast RNA editing of land plants restores conserved amino acid residues at highly specific sites by a C-to-U conversion at the mRNA level [16] and occurs usually at functionally relevant sites [17-21]. The number and location of the editing sites, the so-called editotype [22], varies between different species, but – with the exception of *Marchantia polymorpha *[23] – at least approximately 30 editing sites per plastid chromosome were detected in higher land plants.

Presently, complete plastid genome sequences are available from a huge variety of different organisms [24]. However, the only one for a parasitic plant is that of the achlorophyllous root parasite *E. virginiana *[10]. This genome is presently the smallest known plastid genome of land plants with a size of 70 kb. Despite this reduction in size several typical features for plastid genomes were retained, e.g. the possession of introns and the necessity of RNA editing [25]. Others, such as the possession of a plastid-encoded RNA polymerase (PEP), are absent.

In order to improve knowledge about the capacities of parasitic plants, we sequenced the plastid genomes of *C. reflexa *and *C. gronovii*. Together with the plastid genome of *Epifagus*, this has allowed a comparative analysis of the molecular changes that mark the progression towards holoparasitism and an adaptation to a parasitic lifestyle in land plants.

## Results and discussion

### Size and structure of plastid chromosomes

Sequence data of entire plastid chromosomes were obtained for *C. reflexa *[EMBL: AM711640] and *C. gronovii *[EMBL: AM711639] and compared to two selected known plastid genomes, that of the Solanaceae *Nicotiana tabacum *[26,27] and that of *Epifagus virginiana *[10]. Thus, our data set contains the plastid genome sequences of three parasitic plants and that of *N. tabacum*. The latter species was chosen as non-parasitic reference because it belongs to the same order as *Cuscuta*, Solanales, and its plastid genome has been thoroughly analyzed which is why it has served as reference plant previously [11,12].

In terms of overall size, the plastid chromosome of *C. reflexa *was found to contain 121,521 bp which is very close to the 122 kbp that were estimated based on the extent of hybridization to tobacco [12]. In contrast, the plastid chromosome of *C. gronovii *consists of only 86,744 bp (Table 1) whereas the plastid chromosome size of *E. virginiana *with 70,028 bp is still significantly smaller and remains the smallest sequenced plastid genome of higher land plants known so far [10]. *N. tabacum*, in comparison, possesses a plastid chromosome consisting of 155,939 bp (Table 1) [26,27]. As expected, the genome size reflects the declining dependency on one of the major benefits of plastids, photosynthesis. In comparison with *E. virginiana*, an additional 17 kbp of plastid genome sequence was preserved in *C. gronovii*. This difference is mainly caused by genes encoding the subunits needed for the photosynthetic apparatus, which are missing in *E. virginiana*.

**Table 1:** Properties of the plastid genomes of *Nicotiana tabacum*, *Cuscuta reflexa*, *Cuscuta gronovii *and *Epifagus virginiana*

	*N. tabacum*	*C. reflexa*	*C. gronovii*	*E. virginiana*
total [bp]	155,939	121,521	86,744	70,028
LSC [bp] (% of total)	86,686 (55.6)	79,468 (65.4)	50,973 (58.8)	19,799 (28.3)
SSC [bp] (% of total)	18,571 (11.9)	8,571 (7.0)	7,063 (8.1)	4,759 (6.8)
IR [bp] (% of total)	25,341 (16.3)	16,741 (13.8)	14,354 (16.6)	22,735 (32.5)
% coding	49%	69%	75%	43%
number of genes	113	98	86	40
number of genes with introns (with 2 introns)	18 (3)	12 (3)	5 (1)	4 (2)

**Figure 1:**
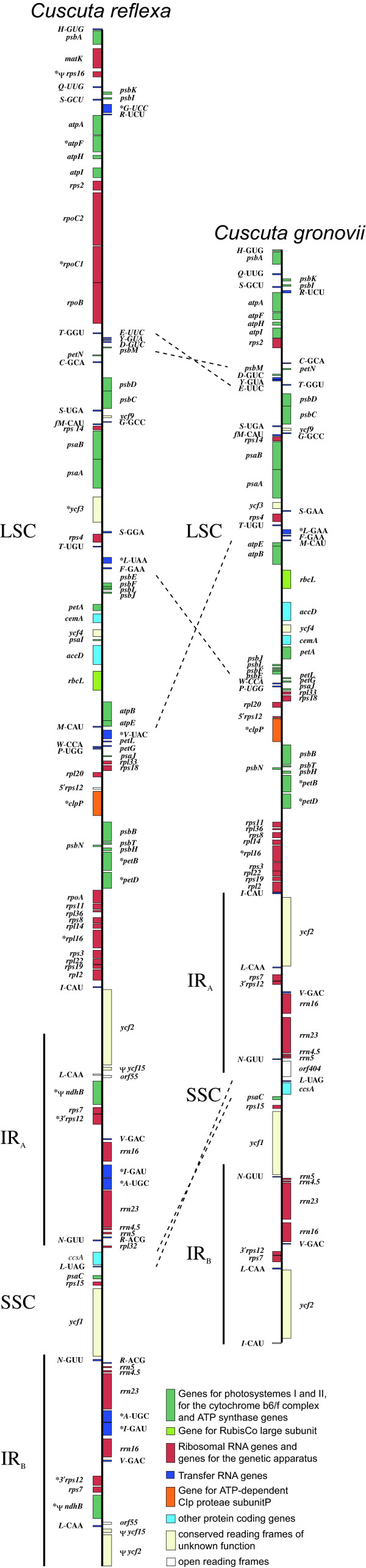
**Gene Maps of the plastid chromosomes of *Cuscuta reflexa *and *Cuscuta gronovii***. Genes shown on the right hand side are transcribed top down and genes on the left hand side bottom up. The large single copy region (LSC) and the small single copy region (SSC) are separated by two inverted repeats (IR_A _and IR_B_). Asterisks indicate intron containing genes. Pseudogenes are marked by Ψ. Dashed lines indicate the inverted regions between *C. reflexa *and *C. gronovii*.

The plastid chromosomes from both *Cuscuta *species show a typical organization with a large single copy region (LSC) and a small single copy region (SSC) separated by two inverted repeat regions (IR_A _and IR_B_) (Table 1; Fig. 1). It should be noted that in contrast to the predicted overall size [12], the predicted sizes of the individual regions of *C. reflexa *were significantly less accurate, with the LSC and SSC being some 21 kbp and 3.5 kbp, respectively, larger than anticipated, while the inverted repeat is roughly 12 kbp smaller than reported by these authors. These substantial deviations demonstrate the value of the more tedious sequence analysis over hybridization analysis.

Interestingly, the IR_A_-LSC junction (J_LA_) in *C. reflexa *was found to be within the *ycf2 *gene. Due to this reduction of the inverted repeat there is only one copy of *rpl2*, *trnI*-cau and one complete *ycf2 *gene. Compared to tobacco and other plastid genomes of higher land plants, *C. reflexa *exhibits three sequence inversions within the plastid chromosome, two in the large single copy region comprising ~2 kb and ~13 kb in length, and one of ~1.5 kb length in the small single copy region. None of these inversions were detected in either *C. gronovii *or *E. virginiana *(Fig. 1). The 13 kb inversion was already hypothesized by Haberhausen *et al. *in 1992 [5]. The same inversion was also observed in another species of the subgenus *Monogyna*, *C. japonica*, but is absent from the subgenera *Grammica *and *Cuscuta *[28]. This is consistent with our findings for *C. gronovii*, which belongs to the subgenus *Grammica*. The two other inversions were also identified only in the plastid genome of *C. reflexa *and may thus imply that the inversions are unrelated to parasitism. For both *Cuscuta *species, overlapping PCR products indicate the existence of a circular form of the plastid chromosomes.

### Coding potential

Both plastid chromosomes of *Cuscuta *encode a reduced amount of genes compared to that of *N. tabacum *(Table 2). Among the genes that are missing in *C. reflexa *are the *ndh *genes that encode for the subunits of the NADH dehydrogenase complex required for chlororespiration. Besides the loss of these genes, the genes *infA*, *trnK*-uuu and the *orf350 *were completely eliminated from the plastid genome, and two ribosomal protein genes (*rpl23*, *rps16*) as well as *ycf15 *were retained only as pseudogenes (Table 2). With the exception of *orf404 *(homologous to the tobacco *orf350*), all genes and pseudogenes mentioned above were also lost in *C. gronovii*. Further specific gene losses on the plastid genome of *C. gronovii *have been detected for *psaI*, *matK, trnV*-uac, *rpl32 *and the *rpo *genes. In addition, there are two tRNA genes whose sequences were completely eliminated from the plastid DNA, and four tRNA genes (*trnA*-ugc, *trnG*-ucc, *trnI*-gau, *trnR*-agc) that have remained only as pseudogenes (Table 2). The lack of some tRNA genes on the plastid genome of the *Cuscuta *species raised the question whether the codon usage was altered in response to the tRNA losses. We therefore performed an analysis of the codon usages in both species. The typically 30 tRNA genes, which are encoded on a ptDNA, are considered to be sufficient to read all 61 sense codons of chloroplast genes [29]. Surprisingly, all 61 sense codons were found in the coding regions of the genes in both *Cuscuta *species and seem to be used, moreover, in a similar proportion as in non-parasitic plants that possess a 'full' plastid tRNA set (Table 3). For example, 77.8% of the lysine residues in tobacco are encoded by the codon AAA, for which tRNA *trnK*-uuu is absent from both *Cuscuta *ptDNAs. For *E. virginiana*, an import of cytosolic tRNAs into the chloroplast was suggested [30,31] which probably must be assumed for *Cuscuta *as well. The mechanism is supposed to be based on the same co-import with protein factors that seems to be responsible for the import of cytosolic tRNAs into mitochondria [32]. However, it is unclear why some tRNAs were retained, whereas others were lost. In this context, it is perhaps noteworthy that the subset of tRNAs conserved in the plastid genomes of parasitic plant plastids (including *Cuscuta*) shows a remarkable overlap with the set of mitochondrial encoded tRNAs for which no import has ever been observed [see also [33]].

**Table 2:** Gene content of *Cuscuta reflexa *and *Cuscuta gronovii *ptDNA compared to *Nicotiana tabacum *and *Epifagus virginiana*

**photosynthetic and chlororespiratory genes**					**ribosomal RNA genes**					**transfer RNA genes**				
	Nt	Cr	Cg	Ev		Nt	Cr	Cg	Ev		Nt	Cr	Cg	EV

*atpA*	+	+	+	Ψ	*rrn16*	+	+	+	+	*trnA*-ugc	+	+	Ψ	Ψ
*atpB*	+	+	+	Ψ	*rrn23*	+	+	+	+	*trnC*-gca	+	+	+	Ψ
*atpE*	+	+	+	-	*rrn4.5*	+	+	+	+	*trnD*-guc	+	+	+	+
*atpF*	+	+	+	-	*rrn5*	+	+	+	+	*trnE*-uuc	+	+	+	+
*atpH*	+	+	+	-						*trnF*-gaa	+	+	+	+
*atpI*	+	+	+	-						*trnfM*-cau	+	+	+	+
*ndhA*	+	-	-	-						*trnG*-gcc	+	+	+	-
										
*ndhB*	+	Ψ	-	Ψ	**RNA polymerase and maturase genes**					*trnG*-ucc	+	+	Ψ	-
										
*ndhC*	+	-	-	-						*trnH*-gug	+	+	+	+
*ndhD*	+	-	-	-						*trnI*-cau	+	+	+	+
*ndhE*	+	-	-	-		Nt	Cr	Cg	Ev	*trnI*-gau	+	+	Ψ	Ψ
										
*ndhF*	+	-	-	-	*matK*	+	+	-	+	*trnK*-uuu	+	-	-	-
*ndhG*	+	-	-	-	*rpoA*	+	+	-	Ψ	*trnL*-caa	+	+	+	+
*ndhH*	+	-	-	-	*rpoB*	+	+	-	-	*trnL*-uaa	+	+	+	-
*ndhI*	+	-	-	-	*rpoC1*	+	+	-	-	*trnL*-uag	+	+	+	+
*ndhJ*	+	-	-	-	*rpoC2*	+	+	-	-	*trnM*-cau	+	+	+	+
*ndhK*	+	-	-	-						*trnN*-guu	+	+	+	+
*petA*	+	+	+	-						*trnP*-ugg	+	+	+	+
										
*petB*	+	+	+	-	**ribosomal protein and initiation factor genes**					*trnQ*-uug	+	+	+	+
										
*petD*	+	+	+	-						*trnR*-acg	+	+	Ψ	+
*petG*	+	+	+	-						*trnR*-ucu	+	+	+	Ψ
*petL*	+	+	+	-		Nt	Cr	Cg	Ev	*trnS*-gcu	+	+	+	+
										
*petN*	+	+	+	-	*infA*	Ψ	-	-	+	*trnS*-gga	+	+	+	Ψ
*psaA*	+	+	+	-	*rpl14*	+	+	+	Ψ	*trnS*-uga	+	+	+	+
*psaB*	+	+	+	-	*rpl16*	+	+	+	+	*trnT*-ggu	+	+	+	-
*psaC*	+	+	+	-	*rpl2*	+	+	+	+	*trnT*-ugu	+	+	+	-
*psaI*	+	+	-	-	*rpl20*	+	+	+	+	*trnV*-gac	+	+	+	-
*psaJ*	+	+	+	-	*rpl22*	+	+	+	-	*trnV*-uac	+	+	-	-
*psbA*	+	+	+	Ψ	*rpl23*	+	Ψ	-	Ψ	*trnW*-cca	+	+	+	+
*psbB*	+	+	+	Ψ	*rp132*	+	+	-	-	*trnY*-gua	+	+	+	+
*psbC*	+	+	+	-	*rpl33*	+	+	+	+					
										
*psbD*	+	+	+	-	*rpl36*	+	+	+	+	**other protein genes**				
										
*psbE*	+	+	+	-	*rps11*	+	+	+	+		Nt	Cr	Cg	Ev
										
*psbF*	+	+	+	-	*rps12*	+	+	+	+	*clpP*	+	+	+	+
*psbH*	+	+	+	-	*rps14*	+	+	+	+	*accD*	+	+	+	+
*psbI*	+	+	+	-	*rps15*	+	+	+	-	*ycf1*	+	+	+	+
*psbJ*	+	+	+	-	*rps16*	+	Ψ	-	-	*ycf2*	+	+	+	+
*psbK*	+	+	+	-	*rps18*	+	+	+	+	*ycf3*	+	+	+	-
*psbL*	+	+	+	-	*rps19*	+	+	+	+	*ycf4*	+	+	+	-
*psbM*	+	+	+	-	*rps2*	+	+	+	+	*ycf5*	+	+	+	-
*psbN*	+	+	+	-	*rps3*	+	+	+	+	*ycf9*	+	+	+	-
*psbT*	+	+	+	-	*rps4*	+	+	+	+	*ycf10*	+	+	+	-
*rbcL*	+	+	+	Ψ	*rps7*	+	+	+	+	*ycf15*	+	Ψ	Ψ	Ψ
					*rps8*	+	+	+	+	*orf350*	+	-	+	+

+: gene present; -: gene deleted; pseudogenes are indicated by Ψ ; Nt: *Nicotiana tabacum*; Cr: *Cuscuta reflexa*; Cg: *Cuscuta gronovii*; Ev: *Epifagus virginiana*

**Table 3:** Codon usages for codons for which the tRNAs are not encoded on the plastid genome of Cuscuta reflexa and Cuscuta gronovii compared to Nicotiana tabacum

Codon (amino acid)	Nt	Cr	Cg
GCT (Ala)	44,52%	43,36%	***40,43%***
GGT (Gly)	34,42%	30,50%	***34,38%***
ATC (Ile)	19,72%	17,66%	***18,10%***
AAA (Lys)	77,81%	***79,21%***	***79,15%***
CGT (Arg)	24,95%	20,87%	***22,67%***
GTA (Val)	38,68%	35,87%	***31,07%***

Bold italic numbers indicate that the tRNA is missing on the ptDNA in the corresponding species. Nt: *Nicotiana tabacum*; Cr: *Cuscuta reflexa*; Cg: *Cuscuta gronovii*

It is apparent that many gene losses from the *Cuscuta *plastid genomes concern genes for the gene expression apparatus such as ribosomal protein genes and tRNA genes but affect also a few genes involved in photosynthetic carbon fixation (*ndh*, *psaI *in *C. gronovii*). The deletion of genes that are typically encoded by the plastid genome in land plants is, however, not a feature that is characteristic for plastid genomes of parasitic plants alone. In *Pinus thunbergii*, for example, the *ndh *genes are not encoded by the plastid genome either [34], while other photosynthetic lineages have lost the *rpl23 *and *rps16 *genes from their plastid DNA. Similar to the tRNA genes, it can, at present, not be ruled out that some or all of these plastid genes have been transferred to the nuclear genome and are imported into the plastids from the cytosol. In fact, this seems to be the case in some non-parasitic plants, for example, with the ribosomal proteins *rpl23 *and *rps16 *which seem to be imported from the nucleus [35-37]. The same situation is discussed for ribosomal proteins of *Epifagus *[31] and is also likely for *Cuscuta *since the detection of photosynthesis-related proteins suggests that plastid translation is functional [2]. So far, a complete gene loss can only be safely assumed for the *rpo *genes of *C. gronovii *where their absence has been confirmed by a genome-wide hybridization [38].

### Promoter structures

In several parasitic plant species, among them *C. gronovii *and *E. virginiana*, the *rpo *genes coding for the PEP-subunits were either truncated or totally deleted from the plastid genome by natural evolution [38-41]. As mentioned above, the existence of a functional nuclear complement of these genes is very unlikely in *C. gronovii*. Transcription in these plastids, therefore, has to rely on an imported NEP or a so far unknown nuclear-encoded RNA polymerase different from that known from angiosperms. In *E. virginiana *all PEP dependent photosynthesis-related genes were eliminated, as well. This is different in *C. gronovii*, which has retained the majority of photosynthesis-related genes despite the loss of the PEP. In conclusion, a nuclear-encoded RNA polymerase has to be responsible for the expression of photosynthesis-related genes at levels sufficient to allow for photosynthesis [42].

**Figure 2:**
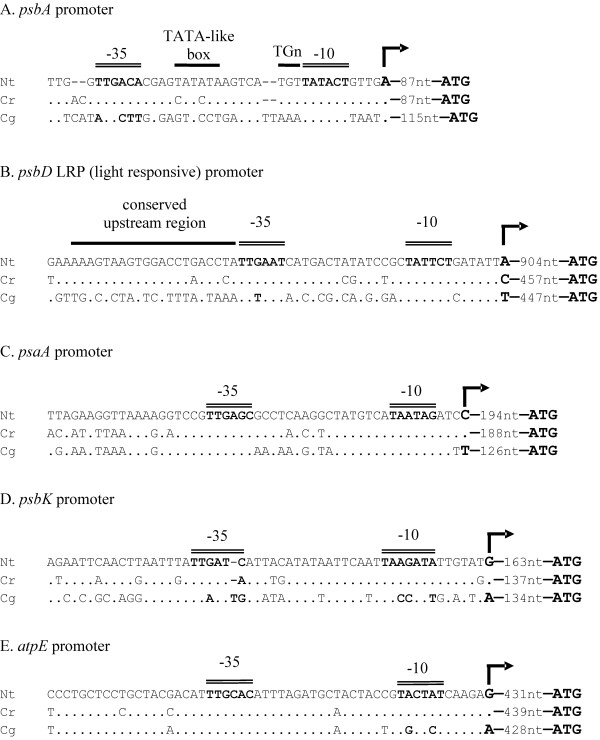
**Comparison of promoter sequences of five PEP promoters in *Nicotiana tabacum*, *Cuscuta reflexa *and *Cuscuta gronovii***. Double lines indicate the consensus motifs of the -10 and -35 boxes typical of plastid PEP promoters. Other conserved regions are marked with a single black line. The distance in nucleotides between the transcription start (indicated by a rightward arrow) and the translation start (ATG) is given. Black dots represent residues that are identical to the nucleotides of *N. tabacum *shown at the top.

In order to investigate what effects this loss of PEP had on the promoters of plastid genes in *C. gronovii*, the 5'-regions of five transcription units known to be transcribed by PEP in non-parasitic land plants were examined (Fig. 2). In tobacco and other photosynthetic plastids, the *psbA *gene is transcribed monocistronically from a single PEP promoter, which is characterized by a TATA-like sequence motif and a TGn motif between the -10 and -35 boxes [43]. While in *C. reflexa *this typical consensus motif is highly conserved, *C. gronovii *exhibits pronounced changes in the sequence leaving only the -10 box unaltered (Fig. 2A). A similar picture emerges with the unique blue-light responsive promoter (LRP) of the *psbD/C *operon (Fig. 2B). This promoter was shown to be activated by high-irradiance blue and UVA light, low temperature, high salt and high osmotic conditions [44,45]. In both *Cuscuta *species this promoter is, however, located closer to the translation start site than in tobacco (Fig. 2B). The promoter of *psbK *[46] shows changes in the -10 and -35 box in *C. gronovii *and only one change in the -35 box of *C. reflexa *(Fig. 2D). In contrast to the three promoters controlling photosystem II genes, the promoter of the *psaA/psaB/rps14 *operon [47] is remarkably conserved not only in *C. reflexa *but also in *C. gronovii *(Fig. 2C). The *atpE *promoter [48] is unaltered in *C. reflexa *as is the -35 box in *C. gronovii*, whereas the -10 box shows two base changes (Fig. 2E).

It was previously shown for the *rbcL *gene, that a shift in transcription start sites accompanied by a replacement of the typical PEP promoter has taken place [42]. The 5' region of the new transcription start site revealed striking similarities to the sequence motifs recognized by the phage-type NEP so that it can be safely assumed that this NEP has taken over *rbcL *transcription in this species. As detailed above, the complete plastid genome sequence of *C. gronovii *has now revealed that other PEP-promoters seem to be significantly altered (Fig. 2), too, so that changes similar to those observed for *rbcL *can be hypothesized and could be part of a systematic and general alteration. As a consequence of these changes, one should expect that major transcriptional regulations such as redox control [49] of the expression of the photosynthetic apparatus are no longer possible.

### Splicing

**Figure 3:**
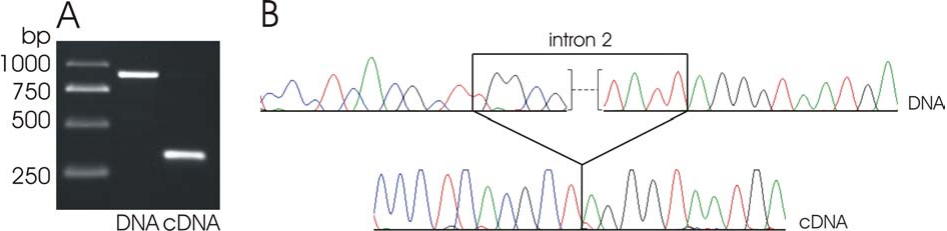
**Splicing of the intron 2 of *clpP *in *Cuscuta gronovii***. A: PCR (DNA) and RT-PCR (cDNA) products of *clpP *overlapping the intron 2 B: DNA and cDNA sequences of the region around the exon/intron 2 boundaries of *clpP*

The *matK *gene, which is coding for a putative maturase that is thought to be essential for the splicing of several plastid introns [50-54], has been lost from the plastid genome of *C. gronovii*. This observation merits attention since *matK *was found on all other sequenced plastid genomes, so far. Therefore, this deletion should be accompanied by changes or losses of the affected introns, unless *matK *was barely transferred to the nuclear genome of *C. gronovii*. The plastid chromosome of tobacco possesses a total of 21 introns in 18 genes. Only one gene possesses a group I intron while the remaining introns belong to the larger group II [15]. The group I intron was retained in both *Cuscuta *species while it was lost from the *Epifagus *ptDNA (Table 4) [10]. Group II introns are divided into group IIA and group IIB introns [15] and splicing of the group IIA introns is postulated to be dependent on the *matK *gene product [50-54]. From the 20 group II introns found in tobacco, eight are of the IIA type. Six of these introns were retained in *C. reflexa*. The two absent group IIA introns in *C. reflexa *are the *rpl2 *intron and an intron in *trnK*-uuu, for which the gene is eliminated in *C. reflexa*. Interestingly, the *matK *gene, that is encoded within the *trnK*-uuu intron in other plastid genomes was retained and is present as a free-standing gene in *C. reflexa*. *C. gronovii *has retained only one group IIA intron belonging to the subgroup IIA1, namely intron 2 of *clpP *(Table 4). Surprisingly, this intron is spliced from the corresponding primary transcript despite the lack of the *matK *gene on the plastid genome (Fig. 3). Therefore, it may either be possible that a MatK-like protein is imported from the cytosol to splice this intron of *clpP *or, alternatively, that this intron does not require the *matK *gene product for splicing. Recently, Hattori *et. al. *[55] could show in the moss *Physcomitrella patens*, that a nuclear-encoded PPR protein is involved in the splicing process of *clpP*. There is also one group IIA intron in *atpF*, for which MatK and the nuclear-encoded factor pCRS1 are necessary for splicing [54,56]. If indeed a different nuclear-encoded factor is responsible for the splicing of the intron 2 of *clpP*, the splicing factor MatK could have been lost completely in *C. gronovii *in adaptation to the parasitic lifestyle. All other group IIA introns known from *Epifagus *or other plastid genomes were eliminated in *C. gronovii *irrespective of the presence of the corresponding gene (Table 4). Among the twelve group IIB introns from tobacco plastid genomes, which are spliced in a *matK*-independent manner, three were lost in *C. reflexa *and seven in *C. gronovii *(Table 4). The presence or absence of all introns and their splicing were confirmed by PCR and RT-PCR (data not shown).

**Table 4:** Appearance of introns in the three parasitic plants *Cuscuta reflexa*, *Cuscuta gronovii *and *Epifagus virginiana*

	Gene (Nt)	*C. reflexa*	*C. gronovii*	*E. virginiana*
group l:				
	*trnL*-uaa	intron	intron	-
				
group ll:				
				
A1	*rps12 *(intron 2)	intron	no intron	intron
	*trnI*-gau	intron	ψ	ψ
	*trnA*-ugc	intron	ψ	ψ
	*trnV*-uac	intron	-	-
	*trnK*-uuu	-	-	-
	*clpP *(intron 2)	intron	intron	intron
				
A2	*atpF*	intron	no intron	-
	*rpl2*	no intron	no intron	intron
				
B1	*petB*	intron	intron	-
	*petD*	intron	intron	-
	*rps16*	-	-	-
	*rpoC1*	intron	-	-
	*ycf3 *(intron 2)	intron	no intron	-
	*clpP *(intron 1)	intron	intron	intron
				
B2	*rps12 *(intron1 *trans*)	intron	intron	intron
	*rpl16*	intron	intron	intron
	*ndhB*	ψ	-	ψ
	*ndhA*	-	-	-
	*ycf3 *(intron 1)	intron	no intron	-
	*trnG*-ucc	intron	ψ	-

All intron containing genes in *N. tabacum *(Nt) are listed. 'intron': intron containing gene; 'no intron': gene without intron; 'Ψ ' indicates pseudogenes; '-': gene not present in the particular plastid chromosome a

### RNA editing

**Figure 4:**
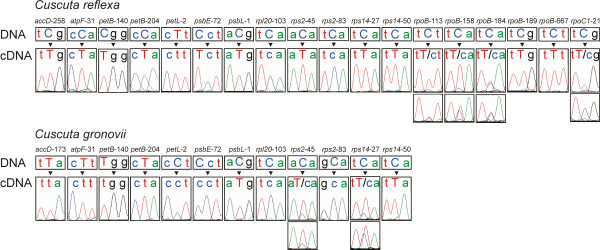
**Sequencing chromatogram excerpts of the editing sites in *Cuscuta***. The uppercase letter indicates the editing site or the conserved amino acid at the DNA level; for partial editing sites two chromatograms are shown in photosynthetic active tissue (top) and in pale tissue (bottom).

To determine the editotypes of *C. reflexa *and *C. gronovii*, we first performed an *in silico *analysis for potential editing sites. All known editing sites in chloroplasts of higher land plants were investigated for their occurrence in *C. reflexa *and *C. gronovii *on the DNA level. All potential editing sites were then analyzed by RT-PCR and sequencing of the cDNAs (Fig. 4). The average amount of editing sites in non-parasitic higher land plants is around 30. 17 potential editing sites were identified in *C. reflexa*, from which eleven were found to be completely edited, four are partially edited and two were found to remain unedited. Taking the gene losses in *C. reflexa *(*ndh *genes) into account, this is in the range of what one would expect and implies *C. reflexa*'s lack of strong selection in its loss of editing sites. Interestingly, the UCA at codon position 103 in *rpl20 *and the TCA at codon position 83 in *rps2 *remain unedited. In other species, these positions are known to be modified through RNA editing such that they encode the highly conserved amino acids. Because the position 103 in *rpl20 *was also found not to be edited in *C. gronovii*, it could be possible that the resulting isoform of Rpl20 with a serine at position 103, is specific for the genus *Cuscuta*. Nonetheless, it cannot be ruled out that this isoform might show an impaired functionality, which can only be tolerated due to the parasitic lifestyle of the genus *Cuscuta*. A different picture emerges for the *rps2*-83 editing site. This editing site remains an unedited GCA alanine codon in *C. reflexa *whereas in *C. gronovii *a TCA codon is found instead, which is also not edited. However, even editing at this position in *C. gronovii *could not restore the conserved leucine. This may indicate that this normally highly conserved position is no longer conserved as a consequence of the parasitic lifestyle of *Cuscuta*.

In contrast, only four out of seven potential editing sites were edited in *C. gronovii*, two of them are partially edited (Table 5 and Fig. 4). For three out of the four partially edited sites in *C. reflexa *and for one partial editing site in *C. gronovii*, we could observe higher editing efficiencies in photosynthetic active tissue (in the tips of the seedlings of *C. reflexa *and *C. gronovii *grown without a host plant; Fig. 4). In contrast to *C. reflexa*, *C. gronovii *shows a pronounced reduction of editing sites compared to other so far investigated angiosperms. On the one hand, this is the result of the loss of the *rpo *genes. On the other hand, the conserved amino acid is already encoded at the DNA level at four sites, namely *accD*-173, *atpF*-31, *petB*-140 and *petB*-204, which makes editing superfluous. In *C. gronovii*, in addition to the *rpl20*-103 and the *rps2*-83 editing sites, two potential editing sites remain unedited at position 72 in *psbE *and position 2 in *petL*, which are edited in *C. reflexa *or already have the conserved amino acid encoded at the DNA level. Moreover, a reduction of the editing efficiency at *rps2*-45 and *rps14*-27 can be seen in *C. gronovii *in comparison to *C. reflexa*. Thus, we speculate that in *C. gronovii *RNA editing might be diminishing with the advanced adaptation to a parasitic lifestyle.

**Table 5:** Editing sites in *Cuscuta reflexa *and *Cuscuta gronovii*

			*C. reflexa*		*C. gronovii*	
gene	pos.	cons.	codon		codon	

*accD*	258/173	L	tCg	S > L	tta	L
*atpF*	31	L	cCa	P > L	ctt	L
*petB*	140	W	Cgg	R > W	tgg	W
	204	L	cCa	P > L	cta	L
*petL*	2	L	ctt	L	cct	P
*psbE*	72	S	Cct	P > S	cct	P
*psbL*	1	M	aCg	T > M	aCg	T > M
*rpl20*	103	L	tca	S	tca	S
*rpoB*	113	L	tCt	S > F*	not encoded	
	158	L	tCa	S > L*		
	184	L	tCa	S > L*		
	189	L	tCg	S > L		
	667	F	tCt	S > F		
*rpoC1*	21	L	tCg	S > L*		
*rps2*	45	I	aCa	T > I	aCa	T > I *
	83	L	tca	S	gca	A
*rps14*	27	L	tCa	S > L	tCa	S > L*
	50	L	tCa	S > L	tCa	S > L

'pos.': amino acid position within the gene; 'cons.': conserved amino acid at this position; upper-case 'C' indicates the editing site; bold indicates that the ratio of edited vs. unedited transcripts depends on photosynthetic activity.

## Conclusion

In the case of phototrophic organisms, parasitism dramatically influences the plant as well as the plastid morphology as seen in the case of *Cuscuta*. Conversely, parasitism is not necessarily reflected by the genome of the plastids as can be observed for the two investigated plastid genomes of *C. reflexa *and *C. gronovii*. Only minor changes are obvious in the plastid genome of *C. reflexa *and the parasitic lifestyle of this plant is therefore not obvious from the structure and coding capacity of the plastid genome. Analysis of plastid gene expression has shown that the relative plastid transcript levels in *C. reflexa *resemble to a high degree those of other parasitic plants [11] so that a facultative adaptation to the parasitic lifestyle has to be proposed. The relative deficiency in change at the genomic level might indicate that this species needs to retain the option of sustaining a host-independent growth for longer periods of time in its natural environment. The high ratio of coding versus non-coding sequence that is characteristic for both *Cuscuta *species that were investigated (see Table 1), might indicate, that an early reaction of the plastid genome to the parasitic lifestyle is a loss of unused and possibly unimportant non-coding parts of the plastid DNA. This essentially results in a condensation to a smaller, more compact chromosome. As the adaptations to parasitism become more pronounced and manifest themselves in organisms with reduced photosynthetic activity like *C. gronovii*, some coding regions of the plastid genome, responsible mainly for plastid gene expression, have become affected. Nevertheless, the capacity to synthesize plastid-encoded subunits of the photosynthetic apparatus is still present, demonstrating an evolutionary pressure to retain photosynthesis-related genes at this stage. It is quite intriguing that the loss of the RNA polymerase genes from the plastid genome, the maturation of the mRNAs and a significant reduction of RNA editing preceded alterations in the components of the photosynthetic apparatus, and might explain the low but nevertheless existent photosynthetic activity of *C. gronovii*. Thus, a step-by-step reduction in the plastid genome may be characteristic for the genus *Cuscuta *and perhaps for all parasitic plants. This can range from mild changes in *C. reflexa*, mainly in the non-coding regions, to massive rearrangements of gene expression in *C. gronovii *to, finally, the loss of all genes for the photosynthetic apparatus as evidenced in *E. virginiana*.

## Methods

### Plant growth

*C. reflexa *and *C. gronovii *were grown in a greenhouse using *Pelargonium zonale *as host plant as described by van der Kooij *et al. *[2] in a light/dark cycle of 16 h/8 h and day and night temperatures of 22 and 18°C, respectively.

### DNA extraction and sequence analysis

Total cellular DNA was extracted by a CTAB-based method [57] or with the DNeasy Plant mini Kit (Qiagen, Hilden, Germany). Sequencing of the plastid chromosome of *C. reflexa *was based on a partial plastid DNA library containing *Bam*HI, *Hind*III, *Pst*I and *Pst*I/*Sal*I fragments. The gaps between the restriction fragments were closed by PCR using Qiagen *Taq *Polymerase and for long range PCR the Long PCR Enzyme Mix (Fermentas, St. Leon-Rot, Germany). A long range PCR approach was used for the plastid chromosome of *C. gronovii*. Amplified PCR products were cleaned up by the PCR clean-up Gel extraction Kit NucleoSpin^® ^Extract II (Macherey-Nagel, Dueren, Germany). Clones and cleaned PCR products were directly sequenced using the DYEnamic ET Terminator Cycle Sequencing Kit (GE-Healthcare, Munich, Germany) on an ABI PRISM^® ^377 DNA sequencer (Applied Biosystems, Darmstadt, Germany). All oligonucleotides used for PCR and sequencing were ordered from MWG Biotech (Ebersberg, Germany). Sequences were edited and assembled using the Sequencher 4.6 (Gene Codes Corporation, Ann Arbor, MI, USA). For the identification of the coding open reading frames the ORF finder and blast tools from NCBI were used. tRNAs were identified with the blast tools from NCBI and the tRNAscan-SE 1.21 [58].

### Analysis of editing sites

RNA was isolated by the CTAB-based method also used for DNA isolation [57]. For cDNA synthesis, the RNA was treated with DNase I and reverse transcribed using Omniscript^® ^Reverse Transcriptase or the OneStep RT-PCR Kit (both Qiagen, Hilden, Germany).

## Abbreviations

bp, base pairs; IR, inverted repeat; LSC, large single copy region; NEP, nuclear-encoded plastid RNA polymerase; PEP, plastid-encoded plastid RNA polymerase; ptDNA, plastid DNA; SSC, small single copy region; *ycf*, hypothetical chloroplast reading frame

## Authors' contributions

HTF performed the sequence analysis of the entire *C. gronovii *plastid genome and parts of the *C. reflexa *plastid genome, annotated the *C. reflexa *and *C. gronovii *plastid genomes, performed the RT-PCR analysis of transcripts (including the identification of splicing and editing sites), did parts of the promoter analysis together with KiK and was involved in drafting the manuscript. SB performed a large part of the sequence analysis of *C. reflexa*. KaK initiated the project and contributed to the work by the interpretation and discussion of the data. UGM participated in the evaluation and interpretation of the data and significantly contributed to the manuscript by critically reviewing it. KiK designed and coordinated the study, provided the plant material, performed the promoter analysis, and, together with HTF, drafted the manuscript. All authors have read and approved of the final version of the manuscript.
